# Boundary-Free Ribosome Compartmentalization by Gene Expression on a Surface

**DOI:** 10.1021/acssynbio.0c00613

**Published:** 2021-02-17

**Authors:** Michael Levy, Reuven Falkovich, Ohad Vonshak, Dan Bracha, Alexandra M. Tayar, Yoshihiro Shimizu, Shirley S. Daube, Roy H. Bar-Ziv

**Affiliations:** † Department of Chemical and Biological Physics, Weizmann Institute of Science, Rehovot 7610001, Israel; ‡ Laboratory for Cell-Free Protein Synthesis, RIKEN Center for Biosystems Dynamics Research, Suita, Osaka 565-0874, Japan; ∥ Department of Biological Engineering, Massachusetts Institute of Technology, Cambridge, Massachusetts 02139, USA; ⊥ Department of Chemical and Biological Engineering, Princeton University, Princeton, NJ 08544, USA; # Department of Physics, University of California, Santa Barbara, Santa Barbara, CA 93106, USA

## Abstract

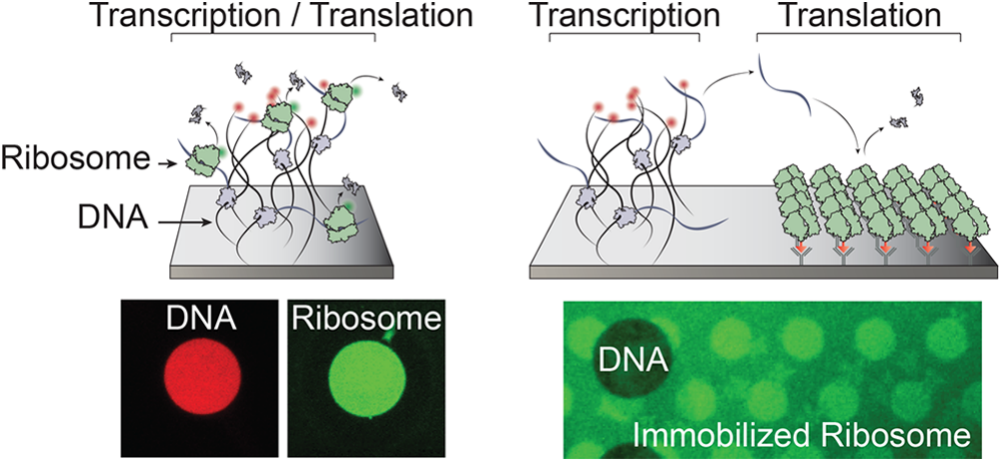

The design of artificial cell models based on minimal surface-bound transcription–translation reactions aims to mimic the compartmentalization facilitated by organelles and inner interfaces in living cells. Dense DNA brushes as localized sources of RNA and proteins serve as synthetic operons that have recently proven useful for the autonomous synthesis and assembly of cellular machines. Here, we studied ribosome compartmentalization in a minimal gene-expression reaction on a surface in contact with a macroscopic reservoir. We first observed the accumulation and colocalization of RNA polymerases, ribosomes, nascent RNAs and proteins, in dense DNA brushes using evanescent field fluorescence, showing transcription–translation coupling in the brush. Fluorescence recovery after photobleaching showed that ribosomes engaged in translation in the brush had a 4-fold slower diffusion constant. In addition, ribosomes in the brush had over a 10-fold higher local concentration relative to free ribosomes, creating a boundary-free functional ribosome-rich compartment. To decouple translation from transcription, we immobilized dense phases of ribosomes next to DNA brushes. We demonstrated that immobilized ribosomes were capable of protein synthesis, forming 2D subcompartments of active ribosome patterns induced and regulated by DNA brush layout of coding and inhibitory genes. Localizing additional molecular components on the surface will further compartmentalize gene-expression reactions.

Cell-free systems that support gene expression reactions are becoming increasingly versatile and efficient for emulating and simplifying cellular processes.^1−5^ Especially powerful is the PURE system made of purified components providing a minimal and controlled environment for RNA transcription and protein translation.^6^ Enriching the PURE system with more functions, gene regulatory elements, and synthetic parts is a promising avenue toward a bottom-up realization of a self-replicating artificial cell.^7−12^ Additional progress has been made in the implementation of the PURE system in microfluidic devices,^13,14^ in encapsulation of reactions in membrane-bound compartments,^15^ or confined to surfaces within silicon compartments.^16^ These approaches expand the capacity to express multiple reaction components and support increasingly complex gene cascaded reactions and networks.

The immobilization of the components that participate in the gene expression reaction adds a spatial element to the reaction, and therefore has a profound effect on reaction outcome. In particular, by anchoring coding genes as DNA brushes and surface traps for assembly intermediates and final products, we recently demonstrated in a PURE gene expression reaction the synthesis and assembly of the five-protein enzyme *E. coli* RNA polymerase (RNAP)^16^ and the small ribosomal subunit (SSU),^10^ composed of 20 ribosomal proteins (r-proteins) and one ribosomal RNA (r-RNA). In addition, we previously observed a localized signal of fluorescently labeled RNAPs and ribosomes at DNA brushes.^14,17^ Taken together, DNA brushes seem to facilitate efficient assembly of protein and RNA complexes through localization of the gene expression machinery. While RNAPs directly bind DNA and their accumulation in DNA brushes is expected, that of ribosomes is intriguing. Could ribosomes, coupled to transcription, penetrate dense DNA phases despite excluded volume interactions?^18^ Are ribosomes in the brush more concentrated than in the bulk solution surrounding the brush? Are nascent proteins released from ribosomes within the brush or only after ribosomes have diffused away from the brush? Can we form patterns of active ribosomes decoupled from transcription?

Here we first characterize the dynamic localization of RNAPs, ribosomes, and nascent RNA and proteins to DNA brushes. We then provide direct evidence that ribosomes are retained within the brush, and not just in its surrounding, with reduced mobility and a 17-fold increased concentration at peak activity, compared to the reaction bulk. These active ribosome compartments lead to protein sources and interaction profiles that could be modulated by the gene fraction within the brush. Finally, we pattern ribosomes directly on the surface surrounding DNA brushes, spatially decoupling translation from transcription, and demonstrate formation of active and inactive ribosome compartments depending on an inhibitory antisense product from the neighboring DNA brushes.

## Results

### A DNA Brush Induces a Propagating Source of RNA and Proteins

**Figure 1:**
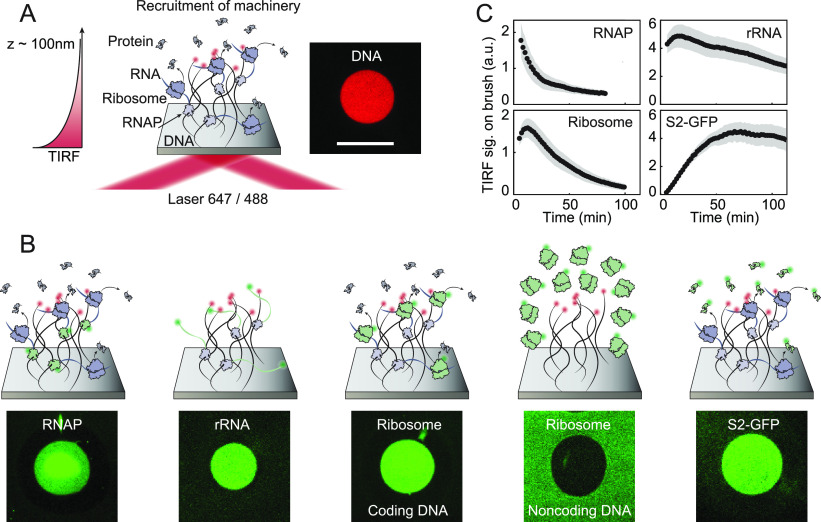
DNA brushes localize gene expression machinery and products. (A) Left: Scheme of TIRF microscopy setup. DNA brush immobilized on a biochip drives spontaneous recruitment of the gene expression machinery and gives rise to a protein source. Right: TIRF Imaging of the DNA brush end-labeled in red (647 nm). Scale bar: 100 μm. (B) Imaging of biomolecules colocalized with active coding brushes in independent experiments. T7 RNA polymerases, *E. coli* ribosomes and r-protein S2 are each fused to GFP (Methods). r-RNA is labeled with Broccoli aptamer. Free ribosomes are recruited to active DNA brushes but excluded from noncoding ones. The labeled species is depicted green above each image. (C) Signal kinetics of the labeled biomolecules measured in TIRF microscopy (independent experiments). Background was subtracted. Time *t* = 0 corresponds to the instance when the temperature crossed 30 °C in its rise from 17 to 37 °C. Error bars are standard deviation of 20–30 brushes.

We studied the effect of DNA immobilization on gene expression reactions by patterning fluorescently labeled DNA brushes with a diameter of 60–80 μm on a fused silica surface coated with a photosensitive and biocompatible polymer monolayer (Figure 1A, Methods). Reactions were enclosed in PDMS chambers of typical dimensions of 1 cm × 1 cm × 150 μm and were initiated by the addition of a minimal gene expression reaction mix and heating the reaction chamber from 17 to 37 °C (Methods). In each experiment a different component of the gene expression reaction was fluorescently labeled, nonoverlapping with the DNA fluorescent label, and we monitored the localization of the labeled species with respect to the DNA brush pattern by total internal reflection fluorescence (TIRF) microscopy (Figure 1A,B, Methods). The DNA brushes coded for a nonfluorescent protein unless indicated otherwise. At any given time, the TIRF signal was proportional to the local concentration of the labeled species in the vicinity of the brush with the decay length of the evanescent excitation ξ ∼ 120 nm.^18^

We first supplemented the gene expression reaction with fluorescently labeled T7 RNA polymerase and observed a rapid increase in TIRF signal within a few minutes after raising the temperature to 37 °C, demonstrating the localization of the RNA polymerase to the brush. The signal, which is proportional to the local rate of transcription, decayed to background levels within 50 min as the gene expression reaction gradually ended due to depletion of nutrients^19^ (Figure 1C). We also detected localization of the RNA product by patterning gene brushes coding for the *E. coli* 16S r-RNA with a Broccoli aptamer^20^ incorporated into its gene sequence, transcribed by a nonlabeled T7 RNA polymerase. The use of a long nontranslatable r-RNA prolongs its localization to the DNA brush and ensures that ribosome binding will not complicate the interpretation. The accumulation and decay of the r-RNA signal, which was the net result of local RNA synthesis and diffusion away from the brush, followed the dynamics of the RNAP signal with a delay of a few minutes, as expected (Figure 1C). The decay of the RNA signal at the brush was slower compared to the RNAP decay, suggesting that some nonspecific physical accumulation of RNA within the DNA brush occurred.

Localization of both the RNAP and nascent RNA to dense DNA brushes was expected due to the direct physical attachment of RNAPs to the DNA during the transcription reaction. Interestingly, we observed the accumulation of fluorescently labeled ribosomes on DNA coding for proteins (coding DNA) and their exclusion from noncoding DNA brushes (no promoter in the sequence). Furthermore, we could directly observe the accumulation of a Green Fluorescent Protein (GFP) fused to a ribosomal protein (S2-GFP) in the brush coding for it (Figure 1B,C). This localization of the translation machinery and product was less expected as for the RNAP and nascent RNA, since transcription and translation are not necessarily coupled in cell-free reactions and ribosomes could bind the mRNA (mRNA) once released from the DNA brush. The localization of ribosomes was previously observed.^14^ Here we measured the accumulation and decay of the ribosome signal, which approximately matched that of the RNA signal. The TIRF signal of nascent protein in the brush increased linearly, saturated at about 50 min, and then decreased marginally and slowly, representing translation in the vicinity of the brush. These observations support the notion that nascent proteins were cotranscriptionally synthesized in the brush by localized ribosomes, with some fraction of them physically adsorbed within the brush while the rest diffused away from the brush.

### Ribosomes Synthesizing Proteins Are Retained in DNA Brushes with Reduced Mobility

**Figure 2:**
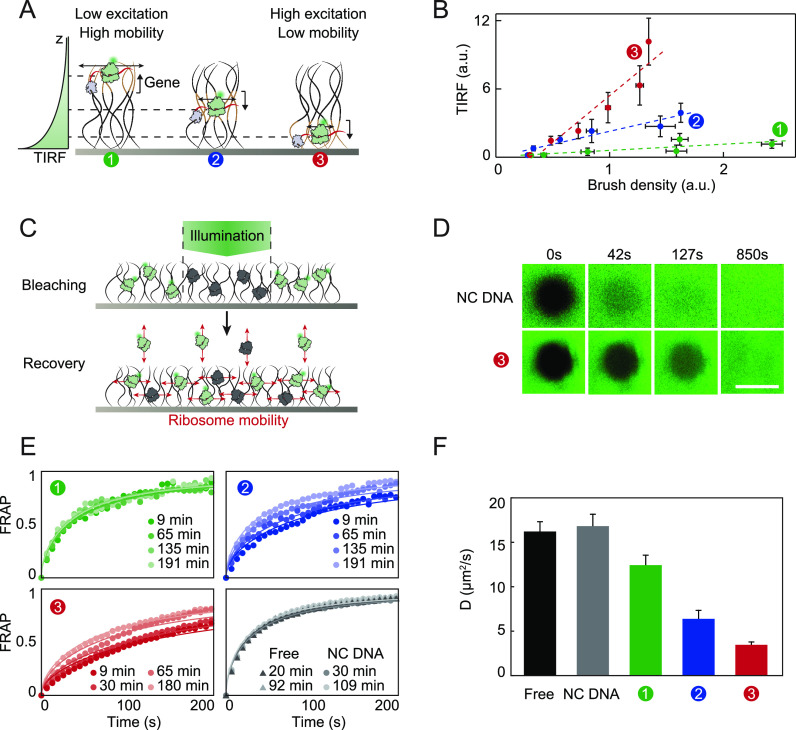
Ribosome reduced mobility within DNA brushes. (A) Scheme of 2.5 kbp DNA brushes with a 400 bp gene located at the top, middle, and bottom of the brush. The promoter upstream of the gene is positioned 1956, 1262, and 687 bp from the tethered end of the DNA and its direction is depicted. The *z* position of ribosomes dictates their mobility and TIRF excitation. (B) TIRF signal for ribosomes localized on the three types of brushes as a function of brush density. Dashed lines are linear fits. Error bars are standard deviation of 3 brushes. (C) Scheme of FRAP experiment on labeled ribosomes (green) in a DNA brush at bleaching (gray) and during recovery. Recovery takes place through ribosome lateral mobility inside the brush and vertical exchange with ribosomes from solution. (D) TIRF images showing fluorescence recovery of labeled ribosomes on a NC brush and on a brush made of DNA from configuration 3 after photobleaching of an 80 μm circular region. Scale bars: 100 μm. (E) Normalized signal recovery after photobleaching at different time points of labeled ribosomes localized on brushes from configurations 1, 2, 3 as well as on NC DNA and free in solution. Solid lines are theoretical fits (Methods). (F) Diffusion coefficients averaged over three measurements in the first 30 min after beginning of gene expression, extracted from the theoretical fits (Methods). Error bars are standard deviation of the three measurements.

We sought to obtain direct evidence for ribosomes localization inside coding DNA brushes. With a typical 1–2 kbp long DNA brush, at a density of about 1000 molecules/μm^2^ and a height of about 100–150 nm under physiological ionic strength, the average distance between DNA molecules was previously measured to be ∼30 nm^21,22^ suggesting that the observed localized ribosomes, with a typical diameter of 21 nm,^23^ might have been excluded from the interior of the DNA brush due to its high density and rather self-organized in a cloud around it, dictated by the high local concentration of nascent mRNA. To identify whether the ribosomes were actually engaged inside of the brush or organized around it, we varied the position of a ∼400 bp gene within 2.5 kbp DNA brushes (Methods, Figure 2A, configurations 1, 2, 3). In configuration 3 and 2 the promoter was directed toward the surface, while in configuration 1 it was pointing outward. Despite the identical sequence of the gene and regulatory elements, the transcription rates in configurations 2 and 3 were previously measured directly to be 2-fold higher than configuration 1, and for all configurations it increased as a function of gene copy number at a fixed brush density.^24^ Indeed, the TIRF signal of labeled ribosomes was responsive to the position and direction of the gene within the brush, with a signal hierarchy of configurations 3 and 2 higher than configuration 1 (Figure 2B). Additionally, the signal of all configurations increased as a function of gene copy number. The sensitivity of the TIRF measurement further revealed a difference between configurations 2 and 3 (Figure 2B, blue and red), suggesting that in each of the three configurations the ribosome distribution in or around the brush was different.

TIRF signals are proportional to ∫ρ(*z*)*e*^–*z*/ξ^ d*z* with ρ(*z*) and *z* the density and distance from the surface of the fluorescent species, respectively, and ξ the decay length of the evanescent excitation,^18^ coupling the density and position of the species. To distinguish between the contribution of ribosome density and distance we performed fluorescence recovery after photobleaching (FRAP) experiments on labeled ribosomes localized on DNA brushes from configuration 1, 2, 3 as well as on noncoding DNA or free in solution (Methods, Figure 2C–F, Figure S1). For each configuration, a large DNA brush with a diameter of about 1 mm was photobleached at well-defined positions, on an 80 μm diameter spot at different time periods after the addition of a PURE reaction supplemented with fluorescently labeled ribosomes. Fluorescence recovery developed through lateral diffusion of ribosomes inside the brush and vertical exchange with ribosomes from solution (Figure 2C). The profiles of the bleached region recovered in a mostly homogeneous way, suggesting that exchange with ribosomes from solution dominated the process (Figure 2D, Figure S1A). The kinetics of recovery were fitted in order to estimate the diffusion coefficient of ribosomes in each configuration and found it to be 16.2 ± 1.1 μm^2^/s, 16.8 ± 1.3 μm^2^/s, 12.4 ± 1.1 μm^2^/s, 6.4 ± 0.9 μm^2^/s, and 3.47 ± 0.32 μm^2^/s for ribosomes free in solution, on noncoding DNA, or on DNA from configuration 1, 2, and 3, respectively (Methods, Figure 2F). These experiments thus revealed that ribosomes in configuration 1, with genes located at the top of the brush, had a similar diffusion coefficient to free ribosomes in solution or above a noncoding DNA brush. In contrast, ribosomes in configuration 3 had the slowest recovery after bleaching, with a diffusion coefficient reduced four to five times. While the kinetics of recovery for configuration 1 and for noncoding DNA were independent of the lag in the bleaching time after the initiation of gene expression, the recovery of ribosomes in configurations 2 and 3 approached that of free ribosomes the longer the lag time, most likely due to the decrease in gene expression rate. Taken together, the TIRF and FRAP measurements are consistent with a scenario of active ribosomes engaged in translation inside the brush while physically bound to RNA molecules cotranscriptionally inside a crowded environment retarding their mobility.

We evaluated the localized ribosome density to be of the order of 450 ribosomes/μm^2^ on a gene-coding brush (Methods, Figure S2), and at a local concentration of about 4500 ribosomes/μm^3^ within the brush volume, which is equivalent to 7 μM (assuming ribosomes were confined inside the brush volume). In these experiments, using fluorescently labeled ribosomes, their bulk concentration was 400 nM (Methods). Therefore, the accumulation of ribosomes to DNA brushes enhanced their concentration by more than an order of magnitude compared to the bulk concentration, only 6-fold lower than the typical ribosome concentration found in bacteria (about 27 000 ribosomes/μm^3^ in *E. coli*).^25^

### Gene Copy Number Determines RNA and Protein Localization Profiles

**Figure 3:**
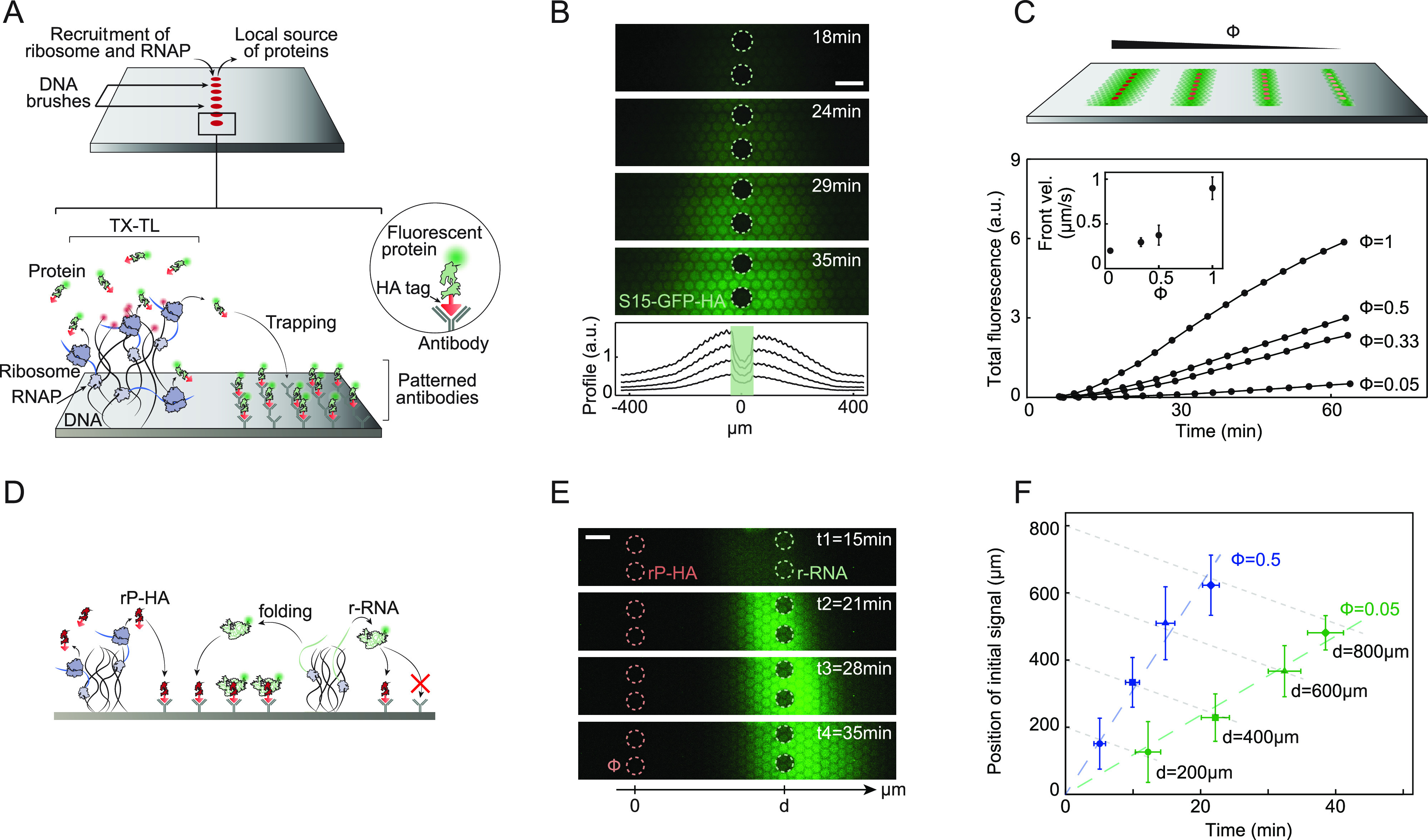
Localized protein sources. (A) Schemes, Top: A line of DNA brushes immobilized on a chip. Bottom: In each brush, transcription and translation colocalize and nascent proteins are trapped on patterned antibodies. (B) Top: Images of the TIRF signal that builds up symmetrically in time on two sides of a line of brushes coding for S15-GFP-HA. Bottom: Profiles of the signal. (C) Top: Scheme of lines of brushes, each with a different gene fraction φ, organized on a single surface 1800 μm apart, generating fluorescent signals of different intensities that were monitored in parallel. Bottom: Total fluorescent signal as a function of time for different gene density φ. Inset: Velocity of the front propagation as a function of gene density φ. (D) Scheme: r-RNA modified with Broccoli aptamer and r-protein S17-HA are synthesized and diffuse from nearby brushes. The binding of r-RNA to surface antibodies is mediated by S17-HA. (E) TIRF images of r-RNA signal buildup in time next to two lines of brushes, as in (D), with the r-protein S17-HA at gene fraction φ = 0.5, separated by 600 μm from the r-RNA brushes. The front of the signal propagates nonsymmetrically toward the right. Scale bar: 100 μm. (F) A space-time plot of the position of first appearance of the r-RNA signal (*Y*-axis) as a function of time (*X*-axis) for different distances *d* between lines of brushes, as defined in (E), with S17-HA brush gene fraction φ = 0.5 (blue) or φ = 0.05 (green). The r-RNA brushes have a fixed gene fraction φ = 0.5. The S17-HA brushes are positioned at the coordinates’ origin. The r-RNA brushes are positioned at a distance *d*, where each dotted line crosses the *Y*-axis.

An important consequence of ribosome localization is the emergence of gene expression profiles, that can influence the dynamics of self-assembly processes^10^ and gene regulation. We studied the concentration profiles of the synthesis products, the nascent RNA and expressed proteins. We first characterized nascent protein profiles by patterning DNA brushes coding for a r-protein fused to GFP and tagged with the high-affinity peptide hemagglutinin (S15-GFP-HA). Anti-HA antibodies were patterned in a hexagonal lattice on the surrounding surface for capture of nascent proteins and sensitive detection of the TIRF signal (Methods, Figure 3A, Figure S3). Identical DNA brushes were organized in lines to obtain a quasi-1D diffusion profile. Upon heating the chamber, a fluorescent signal, dependent on the HA tag, appeared initially next to the line of brushes and propagated laterally and symmetrically on both sides of the source, over hundreds of microns (Figures 3B, S3A). Modeling the space-time profiles as a 1D diffusion process with a continuous point source, we fitted the half-width at half-maximum (HWHM) of the signal (Methods, Figure S3B) and evaluated the diffusion coefficient 67 ± 9 μm^2^/s, consistent with previous evaluations of the diffusion coefficient of GFP.^26^ The experiment was repeated with different gene fractions φ, ranging from 0.02 to 1, at otherwise identical overall DNA density (Methods, Figure 3C). The integrated TIRF signal over the whole pattern was measured in time and found to increase linearly with gene fraction (Figure S3C). To characterize the dynamics of signal propagation, we defined the front of the signal as a threshold value (Methods) and measured its velocity as a function of gene fraction (Figure 3C inset, Figure S3D). The propagation velocity increased with φ, from ∼0.2 to ∼1 μm/s at low and high φ, respectively. The scale of the propagation velocity was determined by the diffusion constant of the synthesized species (Methods). The dependence in φ, however, was a consequence of trapping the diffusing species on antibodies. For low gene density, and consequently low protein concentration, trapping depleted significantly the cloud of proteins, slowing down the propagation of the signal.

To characterize the propagating profile of nascent RNA, we patterned two lines of DNA brushes, one coding for the *E. coli* r-protein S17-HA that has been shown to have fast binding dynamics of 16S r-RNA,^10^ the other coding for the fluorescently labeled 16S r-RNA. The two lines of brushes were separated by a distance *d* = 200, 400, 600, 800 μm and with φ = 0.5 and 0.05 gene fractions of the S17-HA brushes. The r-RNA gene brushes were kept at a fixed brush density and therefore fixed r-RNA production rate (Figure 3D,E, Figure S4D). An r-RNA fluorescent signal propagated on the surface, only in the presence of an HA tag on the r-protein, thereby reflecting r-RNA binding to surface antibodies mediated by S17-HA (Figure S5). Interestingly, signal propagation was nonsymmetrical, much faster to the right of the r-RNA gene brushes than to the left. Since the r-protein source was on the left side of the r-RNA brushes, r-protein concentration was much higher there, saturating the surface antibodies and excess r-protein in solution must have sequestered the binding of the r-RNA to the surface. Indeed, reducing the r-protein gene fraction to φ = 0.02 resulted in a change in the direction of signal propagation toward the left (Figure S4A–C), supporting the notion that excess unbound r-protein determined r-RNA binding to the surface.

We estimated the time and position of first appearance of the signal for every φ and *d* (Figure 3F), and evaluated the propagation speed of the r-protein from the slopes of the dashed lines (Methods, Figure 3F) to be *v* = 0.5 μm/s and 0.2 μm/s for φ = 0.5 and φ = 0.05, respectively. The propagation velocity of S17-HA depended on φ, consistent with the direct observation in Figure 3C. Each dotted line in Figure 3F crosses the *Y*-axis at the position of the r-RNA brush. Therefore, the lines represent the propagation trajectory of the r-RNA from its brush origin to the r-protein on the surface with time. We evaluated the r-RNA propagation speed from the slope of the dotted lines (Methods, Figure 3F) to be *v* = −0.1 μm/s for r-RNA for all the lines, as expected.

### Decoupling Translation from Transcription by Surface-Immobilized Ribosomes

**Figure 4:**
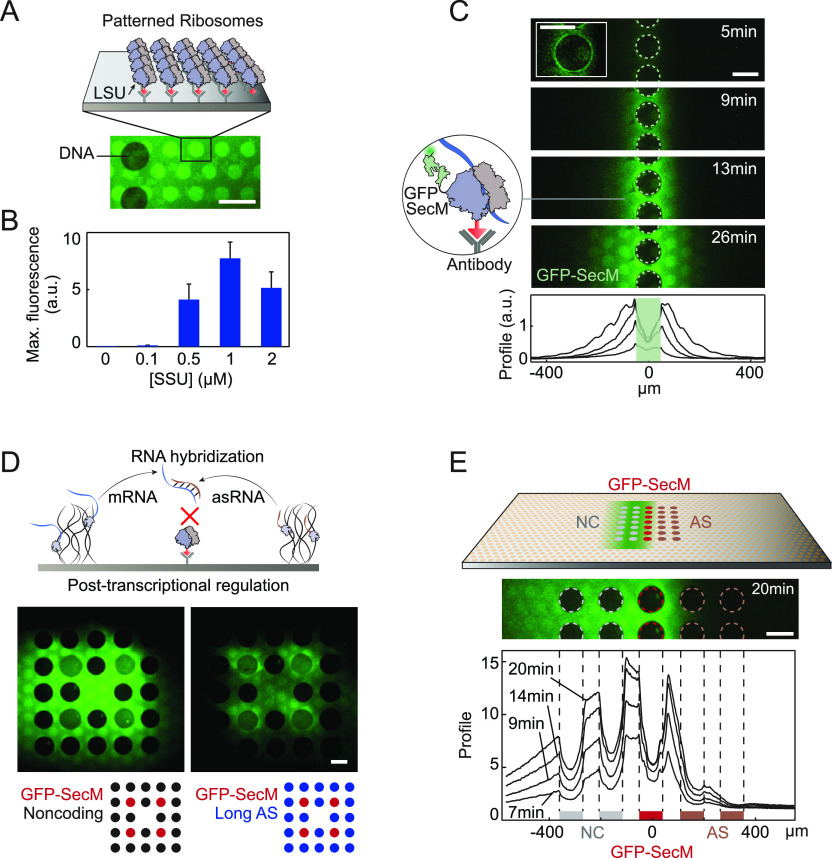
Surface immobilized ribosome carpets. (A) Scheme (top) and TIRF image (bottom) of fluorescently labeled ribosomes immobilized on antibodies next to a DNA brush (dark circles exclude ribosomes) through the large ribosomal subunit (LSU). (B) Maximum GFP-SecM TIRF signal for different concentrations of small ribosomal subunit (SSU) in solution. Error bars are standard deviation of 12 regions. (C) Top: TIRF signal of GFP-SecM builds up symmetrically in time on surface ribosomes next to a line of coding brushes. Bottom: Signal profiles in time. (D) Top: Scheme of post-transcriptional regulation by asRNA hybridizing with GFP-SecM mRNA, inhibiting its translation on surface bound ribosomes. Bottom: TIRF images of maximal GFP-SecM (*t* = 34 min) in the two configurations schematized below. DNA brushes coding for GFP-SecM mRNA (red), asRNA (blue) or NC (black) are depicted as circles with unique arrangements. (E) Top: Scheme and TIRF image of GFP-SecM signal appearing on surface ribosomes around NC brushes but not around AS brushes, on the left and right of a line of GFP-SecM brushes, respectively. Bottom: Profiles of TIRF signal in time. Two configurations with reverse positions for the NC and AS brushes were averaged to avoid artifacts from flow. The positions of the NC (gray), GFP-SecM (red), and AS (brown) lines of brushes are marked. Scale bar: 100 μm.

Inspired by membrane-bound ribosomes in the endoplasmic reticulum of eukaryotic cells, we attempted to immobilize ribosomes on the surface next to DNA brushes prior to initiation of the reaction, decoupling translation from transcription. Toward that, we purified ribosomes with one of the r-proteins in the large subunit fused to GFP-HA and immobilized them in a hexagonal pattern onto anti-HA antibodies (Methods, Figure 4A,B). We evaluated the ribosome density on the surface to be of the order of 300 ribosomes/μm^2^ (Methods), comparable to the *in vivo* value found in the yeast endoplasmic reticulum.^27^

To demonstrate the activity of surface ribosomes, we immobilized DNA brushes encoding GFP with a pause sequence (GFP-SecM)^28^ next to immobilized nonlabeled ribosomes with an HA tag. The pause sequence should delay dissociation of GFP from the ribosomes by about 40 min,^29^ maximizing TIRF detection on surface ribosomes. The chamber was filled with a PURE reaction mix lacking ribosomes. Although surface ribosomes were immobilized under conditions that preserved subunit association (Methods), the addition of purified small ribosomal subunits was required to reach a measurable fluorescent GFP-SecM signal (Figure 4B). By adding an increasing concentration of small subunits to individual chambers organized on the same surface (Figure S6A), we found that 1 μM small subunit was the optimal concentration to achieve maximal signal (Figure 4B). An excess of small subunits presumably saturated the large ribosomal subunit on the surface, precluding association of the mRNA|SSU initiation complex. This optimal small subunit concentration was used in the following experiments.

The GFP-SecM fluorescent signal appeared first as a sharp ring around the brushes, and then propagated symmetrically away from the line of brushes, reflecting diffusion of nascent mRNA from the brush to surface ribosomes, possibly already bound by the ribosomal small subunit (Figure 4C). We measured the dynamics and maximal signal of GFP-SecM on the surface as a function of the gene fraction φ and found that it increased and reached a saturation value at a relatively low density (φ ∼ 0.1), presumably due to the small total amount of ribosomes on the surface (Figure S6B). For all the saturating φ values, the signal increased with time and started reducing after about 30 min due to combined bleaching, release of proteins from ribosomes and end of activity of the gene expression reaction. Applying the 1D diffusion model used above, we fitted the HWHM of the signal (Methods, Figure S6C) and evaluated the diffusion coefficient to be ∼12 ± 2 μm^2^/s. The radius of gyration of a typical mRNA molecule of ∼1000 bp long was measured^30^ to be ∼20 nm implying a theoretical diffusion coefficient in water ∼11 μm^2^/s according to the Stokes–Einstein equation (Methods). Therefore, the signal propagation measured here was consistent with mRNA diffusion.

Having a carpet of ribosomes on the surface, spatially resolved from transcription activity within the brush, we attempted to create compartments of active and inactive ribosomes, governed by the patterns of DNA brushes. In addition to brushes coding for GFP-SecM, we added brushes coding for antisense RNA (asRNA) complementary to regions of the GFP-SecM mRNA in distinct brush patterns (Figure 4D). The asRNA competes with ribosomes on binding to the mRNA, thereby inhibiting translation. Several asRNA were tested (Figure S7): a 1200 bases asRNA complementary to almost the entire GFP-SecM mRNA (“Long AS”), a mixture of two 100 bases asRNAs targeting two regions of the GFP-SecM mRNA including the ribosome binding site (Methods) (“Short AS”) and a 60 bases RNA with no specific affinity to the GFP-SecM mRNA (“Noncomplementary”). The Long AS and Short AS transcripts reduced the signal by 55% and 48%, respectively, compared to a 12% reduction in the case of the noncomplementary transcript, suggesting a sequence specific inhibition. By patterning lines of DNA brushes, a central one coding for GFP-SecM, with Long AS brushes on one side and noncoding brushes on the other side, regions of active and inactive ribosomes were created, surrounding the noncoding and antisense brushes, respectively (Figure 4E, S8).

### Summary

Surface immobilization of DNA and ribosomes in minimal cell-free gene expression reaction offer an opportunity to study the spatial organization of the gene expression machinery and reaction products under conditions of crowding and confinement. The characterization of RNA and protein localized sources and controlled diffusion and interactions provides the basic understanding of the promotion of complex assembly reactions such as the recently demonstrated biogenesis of the *E. coli* small ribosomal subunit.^10^

In a similar scenario to gene expression reactions in the nucleoid of prokaryotic cells, the coupling between transcription and translation was maintained at the immobilized DNA and induced accumulation of RNA and protein products. Using fluorescently labeled ribosomes, we directly showed that ribosomes not only were accumulating in the vicinity of the brush, but also were actually retained within the brush with reduced mobility compared to nontranslating ribosomes. Two mechanisms are possible: either the entire ribosome penetrates the brush to translate cotranscriptionally, or it is only the labeled small subunit which attaches to nascent mRNA, with the resulting translation initiation complex temporarily immobilized on the DNA before associating with large subunit outside the brush. Our demonstration that nascent protein products accumulated inside the brush supports the former, yet direct evidence of labeled large subunit localization in DNA brushes may substantiate it.

The immobilization of ribosomes on surfaces surrounding DNA brushes created a scenario reminiscent of the spatial separation of transcription and translation in eukaryotic cells, providing a delay mechanism that allowed spatial regulation by asRNA and the formation of active and inactive ribosome compartments dictated by the layout of DNA brushes. These capabilities could be utilized in the future to organize complex networks of cascaded reactions based on gene expression regulation in time and space in a bottom-up autonomous fashion. We envision that as more of the gene expression machinery is immobilized on the surface, more opportunities for compartmentalized reactions with increased efficiency could be realized. The physical separation between bound components (old) and newly made ones is valuable for the realization of self-replicating artificial systems.

## Methods

### DNA Preparation

#### Cloning of Genes

Genes of *E. coli* 16S r-RNA and r-protein S15, S17, and S2 were amplified from the genome of *E. coli* K12 JM109 as described in ref (10). The 1542 bp long r-RNA gene, lacking its leader sequence, was cloned in frame with the T7 promoter in the PUREfrex2.0 system control vector (CosmoBio, Japan). Hepatitis delta virus (HDV) ribozyme sequence was inserted at the 3′ end of the 16S r-RNA to ensure formation of an exact 3′ end.^31^ The r-proteins genes were cloned into pIVEX 2.3 (Roche) or in pIVEX 2.5 in frame with a C-terminal HA-tag (THTMVPDYA) as explained in ref (10). The 399 bp long gene coding for the T4 viral protein gp25 under control of the T7 promoter was amplified from the T4 GT7 genome (Nippon Gene, Japan) as described in ref (16). Plasmid coding for GFP-uv3 under a T7 promoter, with a SecM arrest sequence was a kind gift of J. Puglisi.^29^

All fluorescent protein fusions were prepared with the F64L/S65T mutant sequence of enhanced GFP (eGFP). Fusion of r-protein S2-GFP and S15-GFP-HA was prepared by subcloning the eGFP gene at the C-terminus of the r-protein with a seven amino acid long linker (KRAPGTS) between the last amino acid of r-protein and the first amino acid of eGFP. For purification of labeled ribosomes, eGFP was fused at either the C-terminus of r-proteins S2 or L9 with the same linker but in plasmid pRSFduet (Novagen). The HA peptide tag was inserted immediately after the last amino acid of the eGFP gene. For fluorescently labeled T7 RNAP, the eGFP gene was cloned into plasmid pBH161^32^ under control of the *E. coli* UV5 promoter and upstream of the ATG codon of the T7 RNAP gene.

A broccoli aptamer sequence^20^ was cloned in the loop sequence of helix 6 of the *E. coli* 16S r-RNA gene: gtcgaacggtaacaggaagaagctGCGGAGACGGTCGGGTCCAGATATTCGTATCTGTCGAGTAGAGTGTGGGCTCCGCtgcttctttgctgacgagtggc (Capital letters Broccoli aptamer; small letters Helix 6 sequence).

Plasmids encoding asRNA sequences complementary to bases (−61)–41 and to bases 179–270 (with bases 1–3 corresponding to the start codon) of the GFP-SecM gene (Short AS 1 and 2), as well as a 55-bp sequence (gcaagcagcagattacgcgcagaaaaaaaggatctcaagaagatcctttgatc) with similar GC content but no complementarity, were inserted by inverted PCR (iPCR) directly between a T7 promoter and an HDV self-cleaving ribozyme aptamer sequence. Short AS sequences were designed for minimal secondary structure by Mfold Web server^33^ so that the entire self-cleaved transcript, including the initial 5′-G nucleotides of the T7-RNAP transcripts, would be complementary to the GFP-SecM mRNA.

Long AS-coding DNA was assembled by amplifying the GFP-SecM sequence from the plasmid pETC1^29^ and inserting it in reverse orientation under a T7 promoter by Gibson assembly using NEBuilder (New England Biolabs).

#### Preparation of Linear Double-Stranded DNA

Linear double-stranded DNA (dsDNA) fragments were synthesized and conjugated to streptavidin (SA, S0677, Sigma-Aldrich) essentially as described in ref (22) and references therein. Briefly, a 5′-AlexaFluor647 forward primer (F-primer) (IDT) positioned ∼200 bp upstream to the T7 promoter and 5′-biotin reverse primer (R-primer) (IDT) positioned downstream to the T7 terminator were used to amplify genes by PCR with KAPA HiFi HotStart ReadyMix (Kapa Biosystems). Noncoding DNA was prepared similarly to coding DNA but without the T7 promoter.

Biotinylated DNA was conjugated to SA at a 1:1.4 ratio at a final concentration of 150 nM in phosphate buffered saline (1×PBS), supplemented with 7% glycerol to reduce evaporation at the following DNA surface deposition.

#### DNA Configurations for FRAP Experiments

The three DNA configurations described in Figure 2A were amplified from plasmid pIVEX containing the 399 bp long gene of 2.5-gp25^16^ using the following primers.

**Configuration 1**:

F-primer: Biotin-CACGTTAAGGGATTTTGGTC.

R-primer: AlexaFluor647-GATCATGGCGACCACACCCGTC.

**Configuration 2**:

F-primer: AlexaFluor647-GTTGGCCGCAGTGTTATCAC.

R-primer: Biotin-CAATACGCAAACCGCCTCT.

**Configuration 3**:

F-primer: AlexaFluor647-CACGTTAAGGGATTTTG.

R-primer: Biotin-ATCATGGCGACCACACCCGTCC.

The promoter upstream of the 399 bp long gp25 gene was positioned 1956, 1262, and 687 bp from the tethered end of the DNA fragments in configuration 1, 2, and 3, respectively. Note that the biotinylated primer in configuration 1 was the F-primer, while in configuration 2, 3 it was the R-primer, directing transcription in the DNA brush outward for configuration 1, and inward for configuration 2, 3.

#### Preparation of Linear DNA Fragments for GFP-SecM Expression

Linear DNA fragments coding for GFP-uv3 under a T7 promoter, with a SecM arrest sequence were amplified from the plasmid pETC1^29^ using the F-primer Atto647-CATGCAAGGAGATGGCGCC and R-primer Biotin-GTTGGCCGCAGTGTTATCAC.

### Ribosome and RNAP Purification

#### Purification of Fluorescently Labeled T7 RNAP

Preparation of His6-eGFP-T7RNAP was described in a previous work.^32^ Briefly, the fused protein was overexpressed in BL21(DE3) cells after induction with 1 mM IPTG at OD600 of 0.5 for 3 h and first purified by Ni^2+^ affinity chromatography using 2 × 1 mL HisTrap FF columns (GE healthcare). Fractions containing appreciable absorbance at 488 nm, eluted at 90 mM imidazole, were pooled and concentrated using Vivaspin 20 MWCO 10 kDa (Sartorius). Following buffer exchange to 10 mM Tris-HCl pH 7.5; 100 mM NaCl, the sample was loaded onto Superdex200 (GE Healthcare) gel filtration column. Fractions containing appreciable absorbance at 488 nm were pooled and concentrated using Vivaspin 20 MWCO 10 kDa (Sartorius). Purified His6-eGFP-T7RNAP was stored at −80 °C in 50 mM Tris pH 7.5; 100 mM NaCl; 10 mM DTT at a concentration of 50 μM and added to a PUREfrex reaction at a concentration of 700 nM.

#### Purification of Fluorescently Labeled *E. coli* Ribosomes

The ribosome preparation was described in a previous work with some modifications.^34^ Briefly, r-protein fusions S15-GFP (for the brush-coupled ribosomes) and L9-HA or L9-GFP-HA (for unlabeled or labeled surface immobilized ribosomes) under a T7 promoter were overexpressed in *E. coli* (3 h after induction by 1 mM IPTG at OD_600_ of 0.25–0.35). A fraction of the fusion protein was incorporated into ribosomes. After lysis by sonication and french press in ribosome buffer (RB: 10 mM Tris-HCl, 70 mM KCl, 10 mM MgCl_2_, pH ∼ 7.8) the ribosomes were extracted by anion exchange on a quaternary amine column (CIM-multus, QA-8, BIA Separations) in RB + 6 mM β-mercaptoethanol with increasing concentrations of NH_4_Cl. Concentration and buffer exchange (back to RB) of the relevant fractions was done on a 10 kDa MWCO concentration membrane (Sartorius). Batches were diluted to 20–25 μM total ribosomes (as measured by absorption at 260 nm) in RB + 30% glycerol, frozen in liquid N_2_ and stored at −80 °C until use. SDS-PAGE analysis of the purified ribosomes showed no bands corresponding to nonribosomal proteins. Fluorescence measurements of S15-GFP ribosome solution *versus* known eGFP solutions provided an estimate of ∼5–10% of labeled ribosomes among the native *E. coli*. ribosomes.

Purified *E. coli* SSU were a kind gift of A. Yonath. These were purified from native *E. coli* ribosomes by sucrose gradient ultracentrifugation at RB with 1 mM MgCl_2_. When added at 1 μM to a PUREΔR expression reaction of GFP, no detectable amount of protein was produced.

### Chamber Preparation

#### Biochip Prism Mounting and PDMS Chamber Arrangement

The preparation of the chamber was reported in a previous work.^10^ Briefly, fused-silica slides (24 × 24 × 1 mm, UQG Optics) were cleaned in boiling ethanol (10 min) followed by sonication (10 min) and base piranha cleaning (H_2_O_2_:NH_3_:H_2_O; 1:1:4, heated to 70 °C for 10 min). The slides were then coated with a photosensitive and biocompatible polymer monolayer and exposed to 365 nm UV light (2.5 J/cm2) through a custom photomask with an array of hexagons (CAD/Art Services) using UV-KUB (Kloe) to expose reactive amine groups. Biotin *N*-hydroxysuccinimidyl ester (biotin-NHS, Pierce) covalently reacted to UV exposed amine groups resulting in a biotin-patterned surface. The slides were fixed on fused silica prisms (Zell Quarzglas and Technische Keramik). Chambers with typical dimensions of 1 cm × 1 cm were cut in thin PDMS sheets (150 ± 30 μm) and placed on the slides.

#### DNA Deposition

Nanoliter droplets of SA-conjugated linear DNA constructs were deposited in an automated way onto the biotin-patterned surface within the PDMS chamber using GIX Microplotter II (Sonoplot Inc., Middleton, WI) and incubated overnight in a humidity-controlled chamber to allow formation of dense DNA brushes. Before deposition, equimolar solutions of linear DNA constructs were mixed according to the composition of designed brushes. Gene density was tuned by dilution of the solutions (Figure 2B), addition of genes coding for nonfluorescent proteins (Figure 3C) or addition of noncoding DNA (Figure 3E,F and Figure S6B). For brushes with fluorescently labeled DNA, the reported φ is according to epifluorescent signal compared to 100% labeled DNA. Otherwise, φ is given as the molar ratio of the mixture.

In Figure 4E, GFP-SecM genes were mixed with genes coding for a nonfluorescent protein of similar size (T4 bacteriophage gp8) and noncoding DNA in a ratio 5:5:90 to avoid excess of GFP-SecM mRNA and promote competition of the two mRNAs over the limited available translation sites.

#### Antibodies and Ribosome Deposition

Biotinylated Anti-HA-Biotin antibodies (50 mg/mL, High Affinity, 3F10 clone, Roche) were mixed with SA at a molar ratio of 1:1.5 in 1×PBS and incubated 30 min on ice, followed by dilution to 50 nM in PBS before applying them to the chamber. The chamber was washed several times with PBS followed by rinsing with 50 mM Potassium-HEPES buffer pH 7.

For experiments with surface-bound ribosomes, the chamber was washed with Ribosome Buffer (RB) after antibodies deposition, and then replaced by a 2 μM solution of purified ribosomes, of which ∼100–200 nM were estimated to be modified with L9-HA. After further 2-h incubation at 4 °C, the chamber was washed 5 times with RB to remove nonspecifically adsorbed ribosomes.

#### Cell-Free Gene Expression Reactions

The chamber was positioned on a temperature-controlled holder set at 17 °C and placed on an upright microscope (Olympus BX51WI). PUREfrex2.0 cell-free reaction was introduced in the chamber, supplemented with DFHBI-1T (Lucerna, NY) at 60 μM when r-RNA with a Broccoli aptamer was used. Reactions were supplemented with purified ribosomal SSU in experiments involving surface immobilized ribosomes. The chamber was sealed with a glass coverslip and the temperature was then switched to 37 °C to initiate gene expression.

### Imaging

The microscope, positioned on a motorized stage (Scientifica), was equipped with optical filter sets for excitation at 488 and 647 nm and a fluorescent light source (EXFO X-Cite 120Q) to allow epifluorescence microscopy. Total Internal Reflection Fluorescence (TIRF) microscopy was performed by collimating a laser beam (OBIS 488–150 LS) on the side of the prism. Epifluorescence and TIRF images were taken with Andor iXon Ultra camera (Andor Technology PLC., Belfast, UK) and 10× Olympus objective. The stage, the microscope, the lasers and the camera were synchronized on LabVIEW (National Instruments).

### Data Analysis

Images were analyzed with ImageJ and Mathematica 11 (Wolfram Research). Background was subtracted from fluorescent signals unless its value was negligible. In Figure 3C and Figure S3D, the front position was defined as the location where the signal reached twice the background value. In Figure 3F, the position and time of first appearance of the r-RNA signal were defined as the space-time region where the signal had a value between a fourth and a third of the maximum signal reached when *d* = 200 μm. Error bars represent the full region. In this experiment, the positions of first interaction were interpreted as points in space and time reached by the two species. The positions lined up in a consistent way (see dashed and dotted lines in Figure 3F), reflecting propagations from two sources at specific speeds. The dashed and dotted lines in Figure 3F depicted the trajectories of the two species. The propagation speeds were therefore evaluated as the slopes of these lines.

#### Evaluation of Ribosome Density

To evaluate the density of ribosomes localized on an active brush, we imaged them in epifluorescence microscopy in a thin chamber (height: 6 μm) to minimize background from labeled ribosomes in solution (Figure S2). The epifluorescence signal outside of the brush corresponded to the ribosome concentration in solution *C*_0_ = 400 nM. Using the signal of a chamber without ribosomes as a background measurement, we evaluated from the signal on the brush an effective ribosome concentration *C*_eff_ = 523 nM on the brush. The number of ribosomes in the cylinder above the brush *NC*_eff_*VN*_A_, with *V* = *Sh* the volume of the cylinder above the brush, *S* the surface of the brush, *h* the height of the chamber, and *N*_A_ the Avogadro number, was the sum of the number of ribosomes in solution above the brush *C*_0_*VN*_A_ and the number of ribosomes localized on the brush *n*. Here, we assumed the height of the brush (about 100 nm) was negligible compared to the height of the chamber (6 μm). The number of ribosomes localized on the brush was then *n* = *VN*_A_(*C*_eff_ – *C*_0_). We deduced the density of ribosomes per unit surface of the brush:
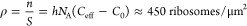
The same method was used to estimate the density of ribosomes immobilized on surface antibodies. In that case, a regular chamber (height: 150 μm) was used because labeled ribosomes containing L9-GFP-HA were only on the surface and not in solution, eliminating the background problem.

#### Simple Model of Diffusion of Synthesized Species from a Line of Brushes

A line of brushes synthesizing proteins is effectively equivalent to a point source in 1D diffusion with the relevant dimension *x* perpendicular to the line. The probability density function for a point source at *x* = 0 is given by
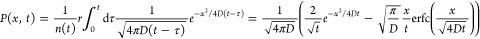
with *n*(*t*) = *rt* the total amount of proteins synthesized after a time *t*, *r* the protein production rate, and *D* the diffusion coefficient of the protein. The mean square displacement is deduced from the probability density function:
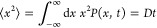
In Figure S3B and S6C, we used the expression  to approximate the half width at half-maximum (HWHM) of the fluorescent signal and fitted the data to evaluate *D*. The actual fitting expression was , where *a* and *b* were added to account for the width of the brush and the delay to initiate translation, respectively. The uncertainty on *D* was calculated from the 99% confidence interval in the fitting procedure.

As a matter of fact, this model describes a situation quite different from the experimental one. Experimentally, we observed the labeled species bound to the surface and not freely diffusing in the volume. The species diffused from a source to a sink, filling up the available sites on the surface. Applying this simple model required the zero-order assumption that the population bound to the surface actually reflected the population in the volume. The assumption was justified by the high affinity between species and traps. It obviously broke down once traps were saturated but until then, it was reasonable to use the model to get rough estimates of diffusion coefficients.

#### Scale of the Propagation Velocity of Synthesized Species from a Line of Brushes

We can estimate the scale of the propagation velocity of the synthesized objects, as measured in Figure 3C, from a line of brushes, in two ways.

The simplest estimate relies on the fact that for a 1D diffusion process, the mean squared displacement obeys: ⟨*x*^2^⟩ = 2*Dt* (the factor 2 should be removed if we consider a 1D point source as derived previously. In any case, this factor is irrelevant when estimating the scale of the process). The measured signal propagates on a distance  in a time *t*, so with an average velocity . Considering that the velocity was measured at *t* ∼ 10 min (see Figure S3D) and with *D* = 67 μm^2^/s as evaluated in the main text, we find *v* ∼ 0.5 μm/s as the velocity scale.

We can also estimate the propagation velocity directly from the probability density function *P*(*x*,*t*) derived previously. To do so, we recall that we defined experimentally the front of the signal as a threshold value, *i.e.*, as a point of constant concentration that propagates in space and time. Let *N*(*x*,*t*) be the number of objects at position *x* and time *t*, then the front propagation obeys d*N*(*x*,*t*) = 0, reflecting the constant number of objects at the front. This condition is equivalent to
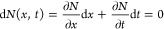
Implying:
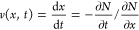
Using the notations introduced previously: *N*(*x*,*t*) = *n*(*t*)*P*(*x*,*t*), leading to
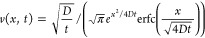
Considering that we measured experimentally the front velocity at *t* ∼ 10 min and *x* ∼ 500 μm (see Figure S3D), and with *D* = 67 μm^2^/s as determined in the main text, we find again *v* ∼ 0.5 μm/s as the velocity scale.

#### Theoretical Diffusion Coefficient for an RNA Molecule in Water

We used the Stokes–Einstein relation to evaluate the diffusion coefficient *D* of a typical 1 kbp RNA molecule in water:
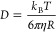
with *k*_B_ the Boltzmann constant, *T* the temperature, *η* the viscosity of water, and *R* the radius of gyration of the RNA molecule.

#### FRAP Experiments

Labeled ribosomes were photobleached using an Ar–Kr laser (Innova 70; Coherent, Inc.) coupled into a single-mode optical fiber (OZ Optics) and focused on the sample through the microscope 10× objective during 15 s. A circular region of about 80 μm diameter was illuminated on a ∼1 mm diameter DNA brush. Recovery was imaged using TIRF microscopy. In Figure 2E, FRAP traces *f*(*t*) were normalized by the final fluorescent value after recovery and fitted to evaluate the diffusion coefficient value using the expression:
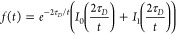
with *τ*_*D*_ = *r*^2^/4*D* the diffusion time scale, *r* = 40 μm the radius of the photobleached region, *D* the diffusion coefficient, *I*_0_ and *I*_1_ the modified Bessel functions.

## Supporting Information Available

Eight figures with supporting schemes, images, and graphs (PDF)
