# Supplementary material for: Boundary-Free Ribosome Compartmentalization by Gene Expression on a Surface
Source: ACS Synth Biol. 2021 Feb 17;10(3):609–19. doi: 10.1021/acssynbio.0c00613 (PMC8023806; doi:10.1021/acssynbio.0c00613)
Supplement: Supplementary file 1 — sb0c00613_si_001.pdf [file sb0c00613_si_001.pdf]

Supporting Information

Boundary-free ribosome compartmentalization by gene expression on a surface

Michael Levy[1,‡], Reuven Falkovich[1, ‡], Ohad Vonshak[1], Dan Bracha[1,#], Alexandra M. Tayar[1,#], Yoshihiro Shimizu[2], Shirley S. Daube[1,*], Roy H. Bar-Ziv[1,*]

[1] *Dept of Chemical and Biological Physics, Weizmann Institute of Science, Rehovot, Israel, 7610001*

[2] *Laboratory for Cell-Free Protein Synthesis, RIKEN Center for Biosystems Dynamics Research, Suita, Osaka 565-0874, Japan*

[*] *Corresponding authors*

[‡] *Equal contribution*

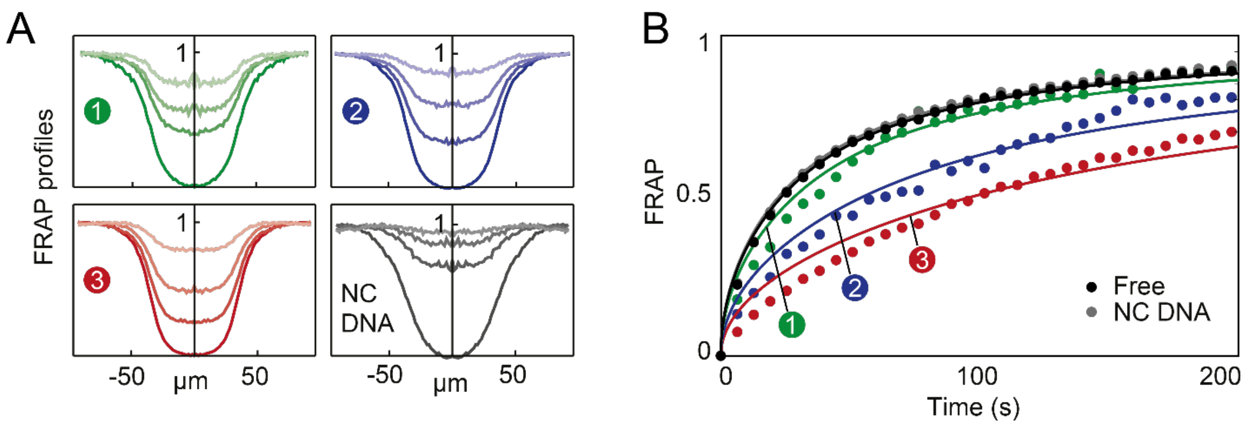

Figure S1. (A) Normalized profiles of the bleached region 0s, 42s, 127s and 424s after photobleaching of labelled ribosomes on the three types of brushes and on a noncoding (NC) brush, at t=20min. (B) Comparison between normalized signals of recovery after photobleaching of labelled ribosomes free in solution, on a NC brush and on the three types of brushes at t=20min.

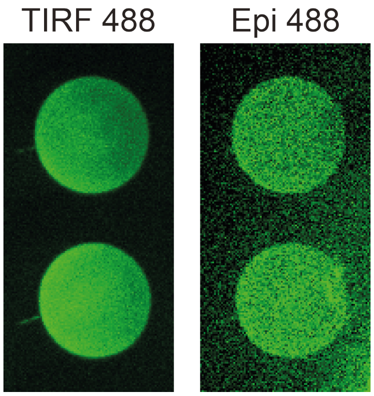

Figure S2. Ribosome localization in thin chamber. Imaging of labelled ribosomes self-organized on DNA brushes in TIRF and epifluorescence microscopy. The brushes are inside a 6μm high chamber to minimize background from nonlocalized labelled ribosomes in epifluorescence microscopy. It enables to evaluate the ribosome density on a brush (Methods).

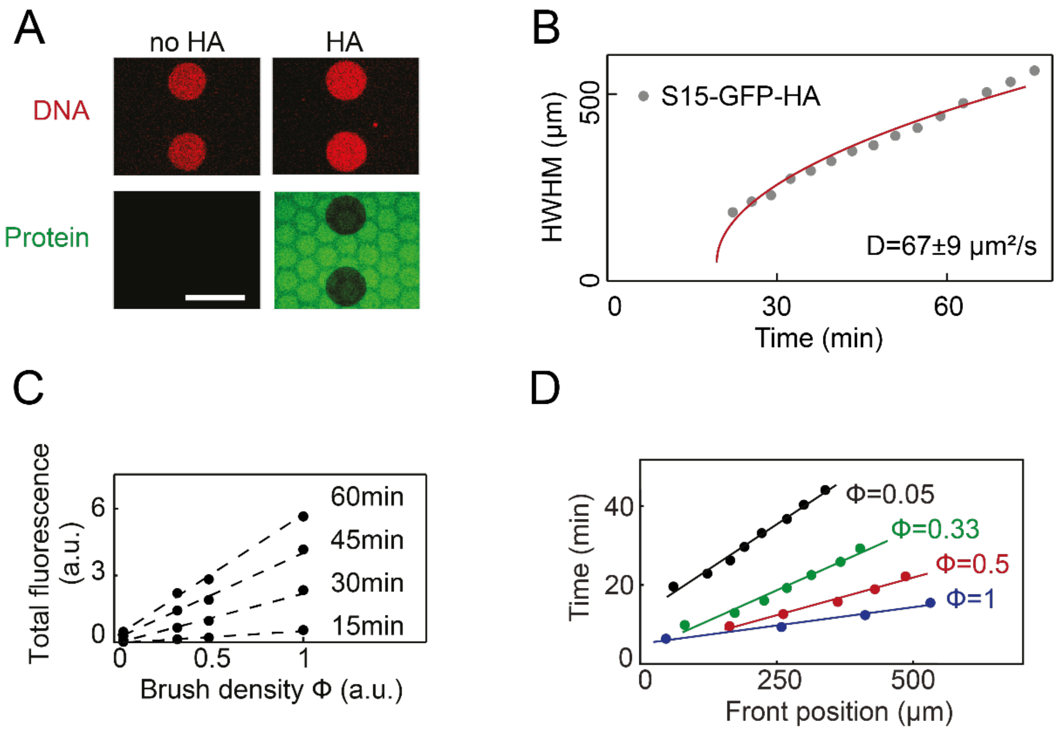

Figure S3. (A) Control experiment to test nonspecific protein adsorption on the surface. Top: Images of end-labeled DNA brushes (red) coding for the ribosomal protein S15-GFP (left) and S15-GFP-HA (right). Antibodies are pattered next to the brushes. Bottom: Corresponding protein signal imaged in TIRF 488 microscopy showing specific binding to anti-HA antibodies. (B) Half width at half maximum (HWHM) of the S15-GFP-HA signal measured on patterned antibodies (see Fig. 3) with theoretical fit (red, Methods). The value for the diffusion coefficient D extracted from the fit is indicated. (C) Total fluorescent signal measured in the experiment presented Fig. 3C. as a function of gene density ϕ at different times. The response is still linear with ϕ after 60min. (D) Position of the front of the signal measured in the experiment presented Fig. 3C. as a function of time. The front velocity displayed in the inset of Fig. 3C. was calculated from the slopes of the linear fits of the data.

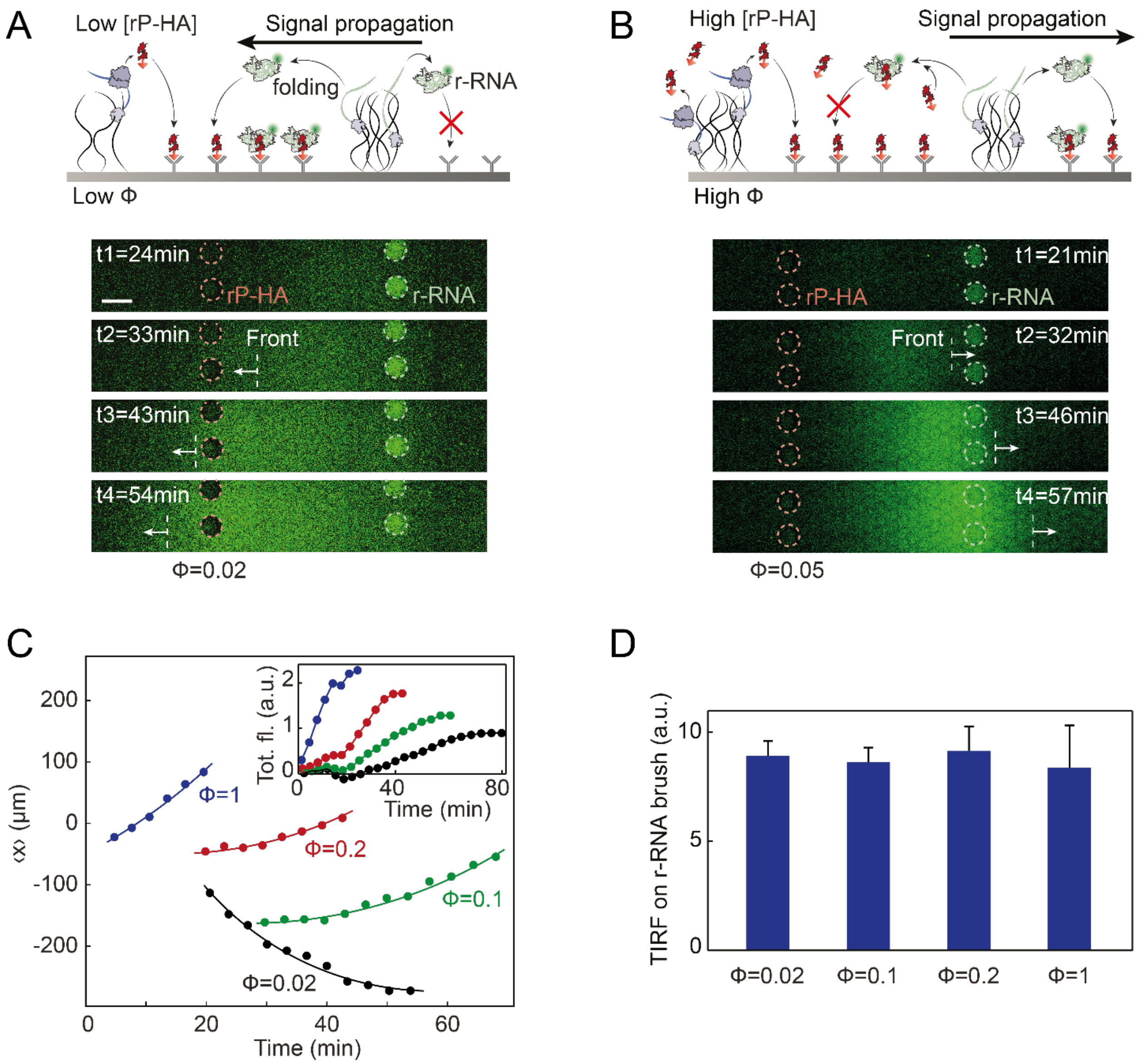

Figure S4. (A,B) Top: Scheme: r-RNA modified with Broccoli aptamer and r-protein S17-HA are synthesized and diffuse from nearby brushes. The binding of r-RNA to surface antibodies is mediated by S17-HA. The direction of propagation of the signal depends on the density ϕ of the rP-HA brush. Bottom: TIRF images of r-RNA signal buildup in time next to two lines of brushes with the r-protein S17-HA at gene fraction ϕ=0.02 (A) and ϕ=0.05 (B), separated by d=600µm from the r-RNA brushes. The front of the signal (white arrows) propagates non-symmetrically toward the left (A) or the right (B) depending on ϕ. Scale bar: 100 µm. (C) Average signal position <x>(t) for d=600µm and different S17-HA gene density ϕ showing the transition between the two directions of propagation. Inset: Total r-RNA fluorescent signal as a function of time for different ϕ. (D) TIRF signal on r-RNA brushes in the experiment presented in Fig. S4C. The brushes had a fixed density and thus presented a constant Broccoli signal. ϕ represents the gene density of the associated S17-HA brushes. Error bars are standard deviation of 5 brushes.

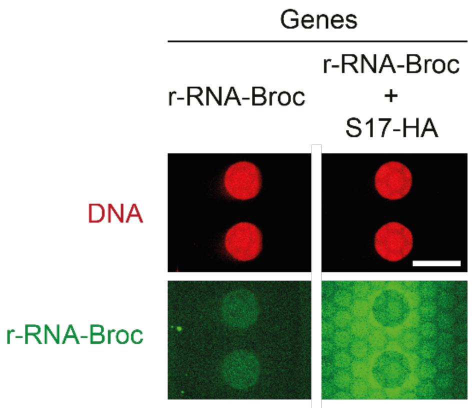

Figure S5. Control experiment to test nonspecific r-RNA-Broc adsorption on the surface. Top: Images of end-labeled DNA brushes (red) coding for r-RNA-Broc alone (left) or with ribosomal protein S17-HA (right). Antibodies are pattered next to the brushes. Bottom: Corresponding r-RNA-Broc signal imaged in TIRF 488 microscopy showing specific binding conditional to the presence of S17-HA.

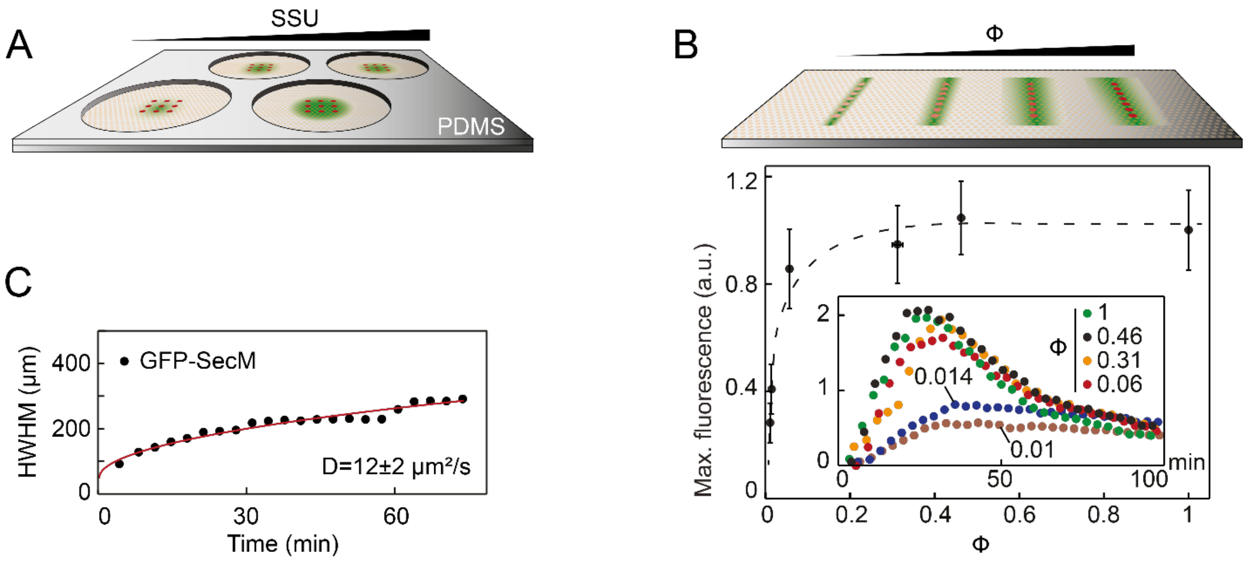

Figure S6. (A) Scheme: GFP-SecM brushes in independent 3mm PDMS chambers to test the effect of different SSU concentrations supplemented in solution (see Fig. 4B). (B) Top: Scheme: lines of brushes with different GFP-SecM gene density ϕ organized on a surface covered with patterned ribosomes, generating fluorescent signals of different intensities. Bottom: Maximum signal as a function of gene density ϕ. Dashed line is guide for the eye. Error bars are standard deviation of 7-8 regions next to the line. Inset: GFP-SecM signal as a function of time for different values of ϕ. (C) Half width at half maximum (HWHM) of the GFP-SecM signal measured on patterned ribosomes (see Fig. 4C) with theoretical fit (red, Methods). The value for the diffusion coefficient D extracted from the fit is indicated.

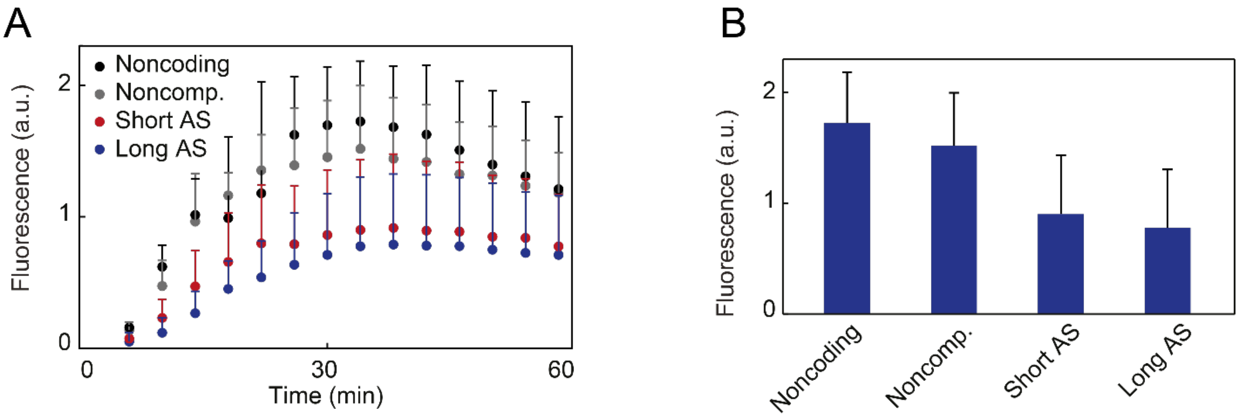

Figure S7. Post-transcriptional regulation with asRNA. (A) GFP-SecM signal as a function of time in the configurations described in Fig. 4D and designated as “Noncoding”, “Noncomplementary”, “Short AS” and “Long AS” in the main text. (B) Histogram reporting a timepoint of the kinetics presented in Fig. S7A at t=34min corresponding to signal maximum. Error bars are standard deviation of 4-7 repeats.

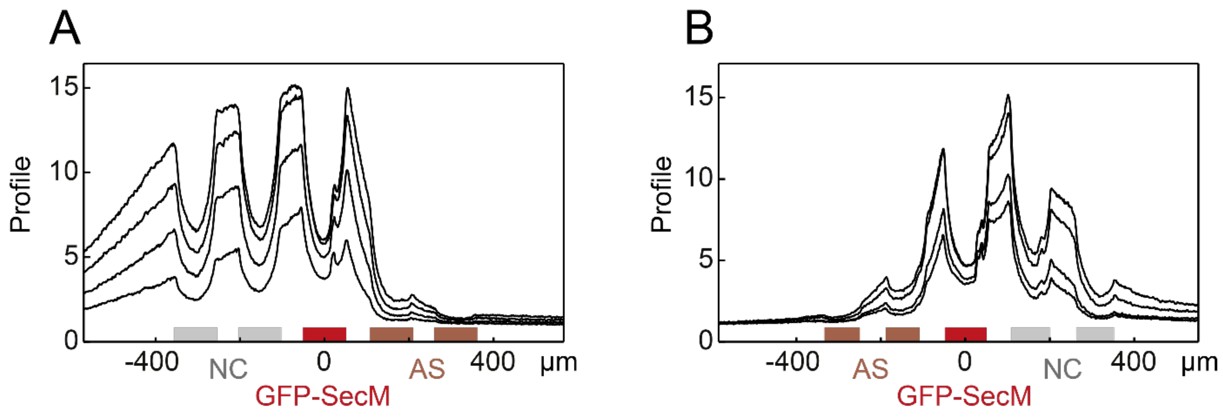

Figure S8. Profiles of TIRF signals for a line of GFP-SecM brushes surrounded by asRNA brushes on one side and noncoding (NC) brushes on the other side at time t=7, 9, 14 and 20min. (A) and (B) present two inverted configurations to avoid artefacts from flow. (A) and (B) were averaged in Fig. 4E after inverting (B).
